# Supplementary material for: Involvement of an orphan response regulator of the two-component regulatory system in the formation of physiologically mature sporangia in Actinoplanes missouriensis
Source: Microbiol Spectr. 2025 Feb 27;13(4):e03272-24. doi: 10.1128/spectrum.03272-24 (PMC11960193; doi:10.1128/spectrum.03272-24)
Supplement: Supplemental figures and tables — Figures S1 to S8; Tables S1 and S2. [file spectrum.03272-24-s0001.pdf]

Involvement of an orphan response regulator of the two-component regulatory system in the formation of physiologically mature sporangia in *Actinoplanes missouriensis*

Takuya Akutsu,[1] Zhuwen Tan,[1] Aiko Hirata,[2] Takeaki Tezuka,[1,3,*] Yasuo Ohnishi[1,3,*]

[1]Department of Biotechnology, Graduate School of Agricultural and Life Sciences, The University of Tokyo, Bunkyo-ku, Tokyo, Japan

[2]Bioimaging Center, Graduate School of Frontier Sciences, The University of Tokyo, Kashiwa-shi, Chiba, Japan

[3]Collaborative Research Institute for Innovative Microbiology, The University of Tokyo, Bunkyo-ku, Tokyo, Japan

*Address correspondence to Takeaki Tezuka, atezuka@mail.ecc.u-tokyo.ac.jp; Yasuo Ohnishi, ayasuo@mail.ecc.u-tokyo.ac.jp

Contents

Fig. S1. Construction of the Δ*asfR* strain

Fig. S2. Alignment of amino acid sequences of AsfR and its homologs

Fig. S3. Transcript levels of *asfR*

Fig. S4. Observation of sporangium dehiscence using phase-contrast microscopy

Fig. S5. Bacterial two-hybrid assay of AsfR and 33 HKs

Fig. S6. SEM observation of sporangia and mycelia produced on HAT agar after 7 days of cultivation

Fig. S7. Observation of sporangium dehiscence in the wild-type and deletion mutant strains of HK genes

Fig. S8. Number of spores released from sporangia in the wild-type and deletion mutant strains of HK genes

Table S1. Primers used in this study

Table S2. Putative sensor HK genes on the *A. missouriensis* genome

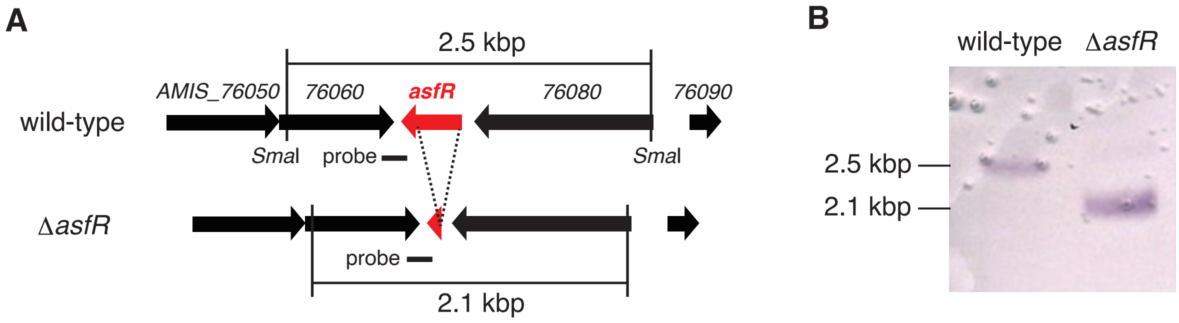

Fig. S1. Construction of the Δ*asfR* strain. (A) Gene organization around *asfR*. (B) Southern blotting using SmaI-digested genomic DNAs of the wild-type and Δ*asfR* strains. The probe position is shown in (A).

A

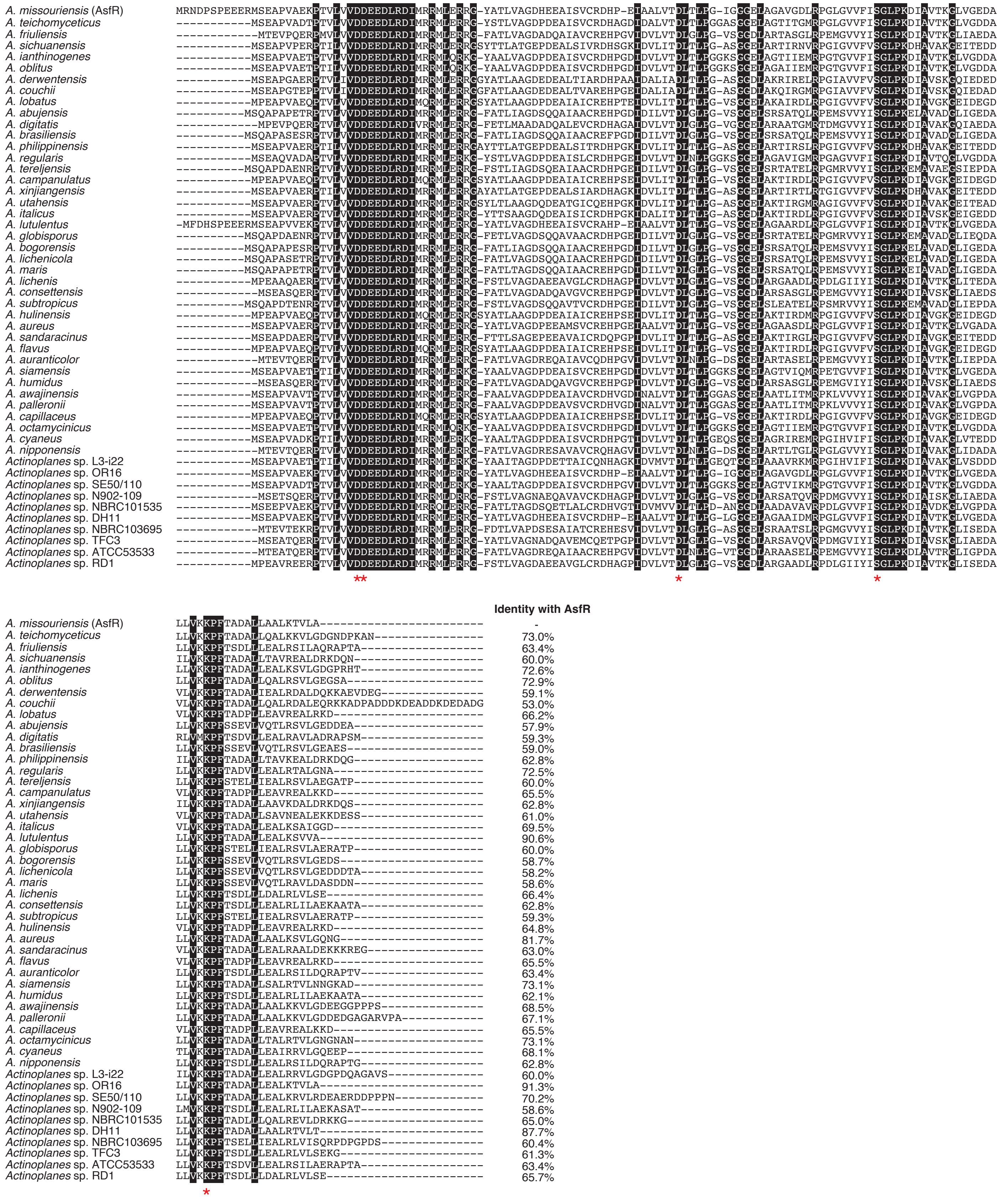

Identity with AsfR
A. missouriensis (AsfR) -
A. teichomyceticus 73.0%
A. friuliensis 63.4%
A. sichuanensis 60.0%
A. ianthinogenes 72.6%
A. oblitus 72.9%
A. derwentensis 59.1%
A. couchii 53.0%
A. lobatus 66.2%
A. abujensis 57.9%
A. digitatis 59.3%
A. brasiliensis 59.0%
A. philippinensis 62.8%
A. regularis 72.5%
A. tereljensis 60.0%
A. campanulatus 65.5%
A. xinjiangensis 62.8%
A. utahensis 61.0%
A. italicus 69.5%
A. lutulentus 90.6%
A. globisporus 60.0%
A. bogorensis 58.7%
A. lichenicola 58.2%
A. maris 58.6%
A. lichenis 66.4%
A. consettensis 62.8%
A. subtropicus 59.3%
A. hulinensis 64.8%
A. aureus 81.7%
A. sandaracinus 63.0%
A. flavus 65.5%
A. auranticolor 63.4%
A. siamensis 73.1%
A. humidus 62.1%
A. awajinensis 68.5%
A. palleronii 67.1%
A. capillaceus 65.5%
A. octamycinicus 73.1%
A. cyaneus 68.1%
A. nipponensis 62.8%
Actinoplanes sp. L3-i22 60.0%
Actinoplanes sp. OR16 91.3%
Actinoplanes sp. SE50/110 70.2%
Actinoplanes sp. N902-109 58.6%
Actinoplanes sp. NBRC101535 65.0%
Actinoplanes sp. DH11 87.7%
Actinoplanes sp. NBRC103695 60.4%
Actinoplanes sp. TFC3 61.3%
Actinoplanes sp. ATCC53533 63.4%
Actinoplanes sp. RD1 65.7%

B

```
A. missouriensis (AsfR)      MRNDPSPEEERMSEAPVAEKPTVLVVDDEEDLRDIMRRMLERRGYATLVAGDHEEAISVCRDHP-EIAALVTDLTLPGIGGGELAGAVGDLRPGLGVVFISGLPKDIAVTKGLVGEDA
Patulibacter sp. NPD049589   -----------MSEAPVAERPTVLVVDDEEDLRDIMRRMLERRGYATLVAGDPEEAISVCREHPGEIAALVTDLTLPGASGGELAGAASDLRPGLGVVFISGLPKDIAVTKGLVGADA
P. ferrugineum               ------------MTEVPEERPTVLVVDDEEDLRDIMRRMLERRGFATLVAGDSDQALTVCREHPGDIDVLVTDLGLPGVSGGEMARTCTQLRPDMGVVYISGLPKDIAVTKGLINEDS
C. caeruleus                 ------------MSESTQERPTVLVVDDEDDLRDIMRRMLERRGFATLIAGDADQALAVCRDHPGDIDVLVTDLTLPDVPGGDVARDCTQMRPGMGVVFISGLPKDMAVAKGLIDEDS
N. flavum                    ------------MSEDQRERPTVLVVDDEEDLRDIMRRMLERRGYATLVAGDADQAIAVCQDHPGPIAVLVTDLGLPGVSGGEMADRCRELRPDMGVVYISGLPKDIAVSKGLIPQDA
S. yamanashiensis            -------------MSEEADRPTVLVVDDEEDLRDIMRRMLERKGFRALVAGDSEEAVAACRTHTGDIDVLLTDLGLPGVSGGDLAREAATLRPGMRVVYISGLPREVALDKGLIAADA
K. cinnamomea                -------------MTEAPAQPTVLVVDDEEDLRDIMRRMLERRGFRALVAGNSEQALEICRSQDGAIDILVTDLGLPGVSGGELAHAAAELRPTMAVVYVSGLLKEIAVDQGLIPGDA
P. lichenicola               ----------MSQAPASETRPTVLVVDDEEDLRDIMRRMLERRGFATLVAGDSQQAIAACREHPGDIDILVTDLGLPGVSGGELSRSATQLRPEMSVVYISGLPKELAVADGLIGEDA
S. lichenis                  -----------MPEAAQAERPTVLVVDDEEDLRDIMRRMLERRGFSTLVAGDAEEAVGLCRDHAGPIDVLVTDLGLPGVSGGDLARGAADLRPDLGIIYISGLPKDIAVTKGLITEDA
W. consettensis              ------------MSEASQERPTVLVVDDEEDLRDIMRRMLERRGFATLVAGDADQAVGVCREHPGPIDVLVTDLGLPGVSGGDLARSASGLRPEMGVVYISGLPKDIAVSKGLIAEDS
                                                    **                               *                              *
```

```
                                                                     Identity with AsfR
A. missouriensis (AsfR)      LLVKKPFTADALLAALKTVLA-------            -
Patulibacter sp. NPD049589   LLVKKPFTADALLEALKAVLSQEG----          81.7%
P. ferrugineum               LLVKKPFTADLLLEALRVILAEKAPTT-          63.4%
C. caeruleus                 VLVKKPFTSELLLGALRSMLAEKAPTT-          59.3%
N. flavum                    LLVKKPFTSDLLLEALRLVLAEAANT--          65.3%
S. yamanashiensis            RLVKKPFTSDALVTSLREALAAPATAP-          55.2%
K. cinnamomea                VLIKKPFTSDVLIDTLHTVISGEAPSTA          53.4%
P. lichenicola               LLVKKPFSSEVLVQTLRSVLGEDDDTA-          58.2%
S. lichenis                  LLVKKPFTSDLLLDALRLVLSE------          66.4%
W. consettensis              LLVKKPFTSDLLLEALRLILAEKAATA-          62.8%
                                 *
```

Fig. S2. Alignment of amino acid sequences of AsfR and its homologs. (A) Alignment of AsfR and its homologs from 50 *Actinoplanes* bacteria, including *A. missouriensis*. All sequences exhibited a similarity of at least 53.0% identity. (B) Alignment of AsfR and its homologs in nine bacteria (*Patulibacter* sp. NPD 049589, *Pseudosporangium ferrugineum*, *Couchioplanes caeruleus*, *Nucisporomicrobium flavum*, *Spirilliplanes yamanashiensis*, *Krasilnikovia cinnamomea*, *Paractinoplanes* lichenicola, *Symbioplanes lichenis*, and *Winogradskya consettensis*). All sequences exhibited at least 53.4% identity. In A and B, five residues that are highly conserved among the RR receiver domains are indicated by red asterisks. Identical amino acid residues are shown in black background. The similarity of the amino acid sequence of each protein with that of AsfR is shown at the end of the alignment.

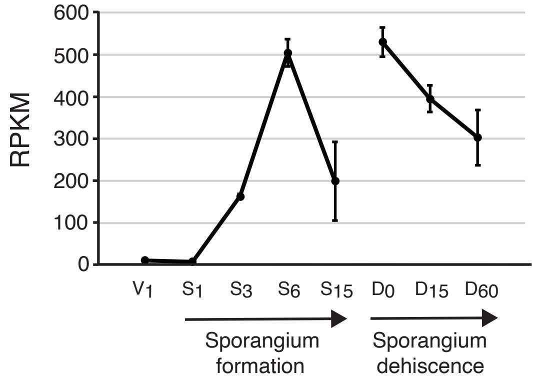

Fig. S3. Transcript levels of *asfR*. Transcripts were examined using RNA-Seq analysis under various culture conditions. RNA samples were prepared from substrate hyphae grown on YBNM agar for 1 day (V_1), substrate hyphae or mixtures of substrate hyphae and sporangia grown on HAT agar for 1, 3, 6, and 15 days (S_1, S_3, S_6, and S_{15}, respectively), and sporangia (including some substrate hyphae) incubated in 25 mM histidine solution to induce sporangium dehiscence for 0, 15, and 60 min (D_0, D_{15}, and D_{60}, respectively). The average number of reads per kilobase of coding sequence per million mapped reads (RPKM) values ± standard errors from three biological replicates are shown.

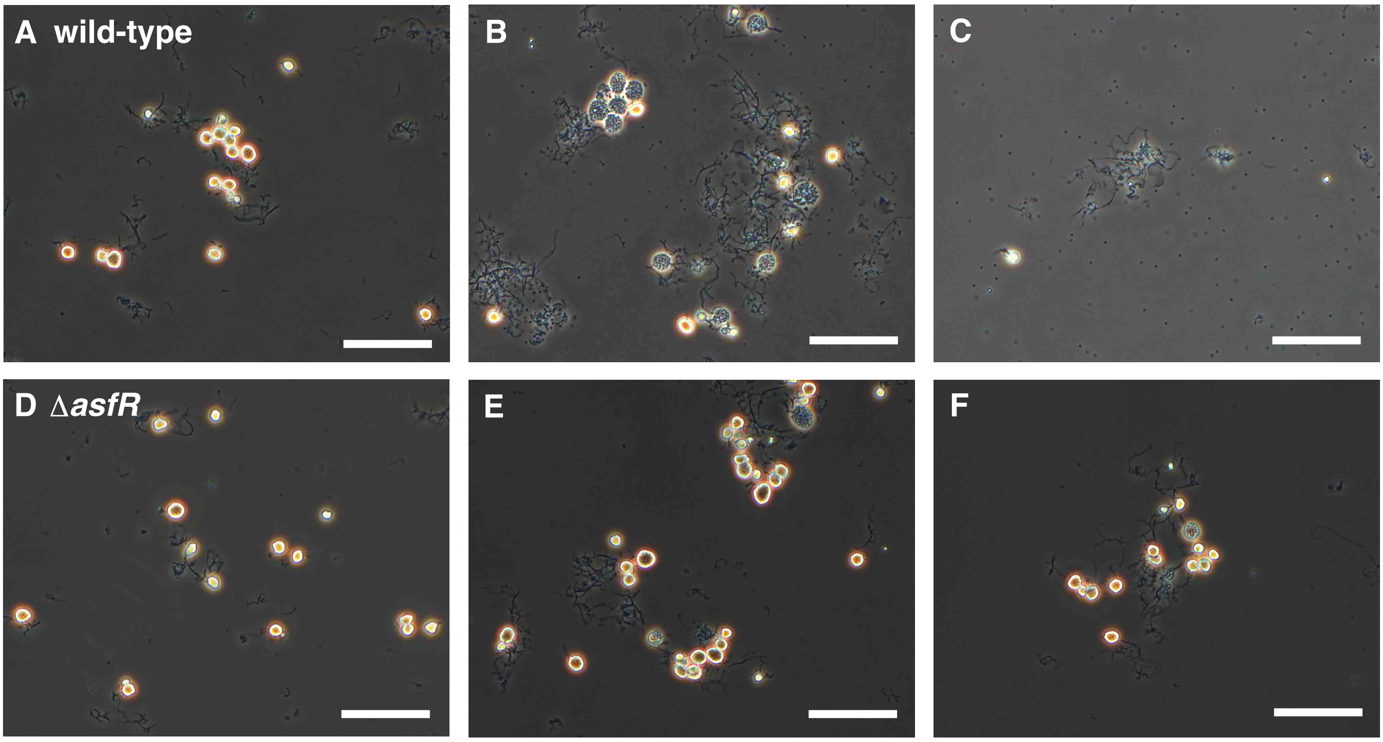

Fig. S4. Observation of sporangium dehiscence using phase-contrast microscopy. Sporangia produced on HAT agar were harvested and suspended in 25 mM histidine solution to induce sporangium dehiscence. Panels A–F are the entire images of the microscopic fields shown in Fig. 3A–F. Scale bars, 50 μm.

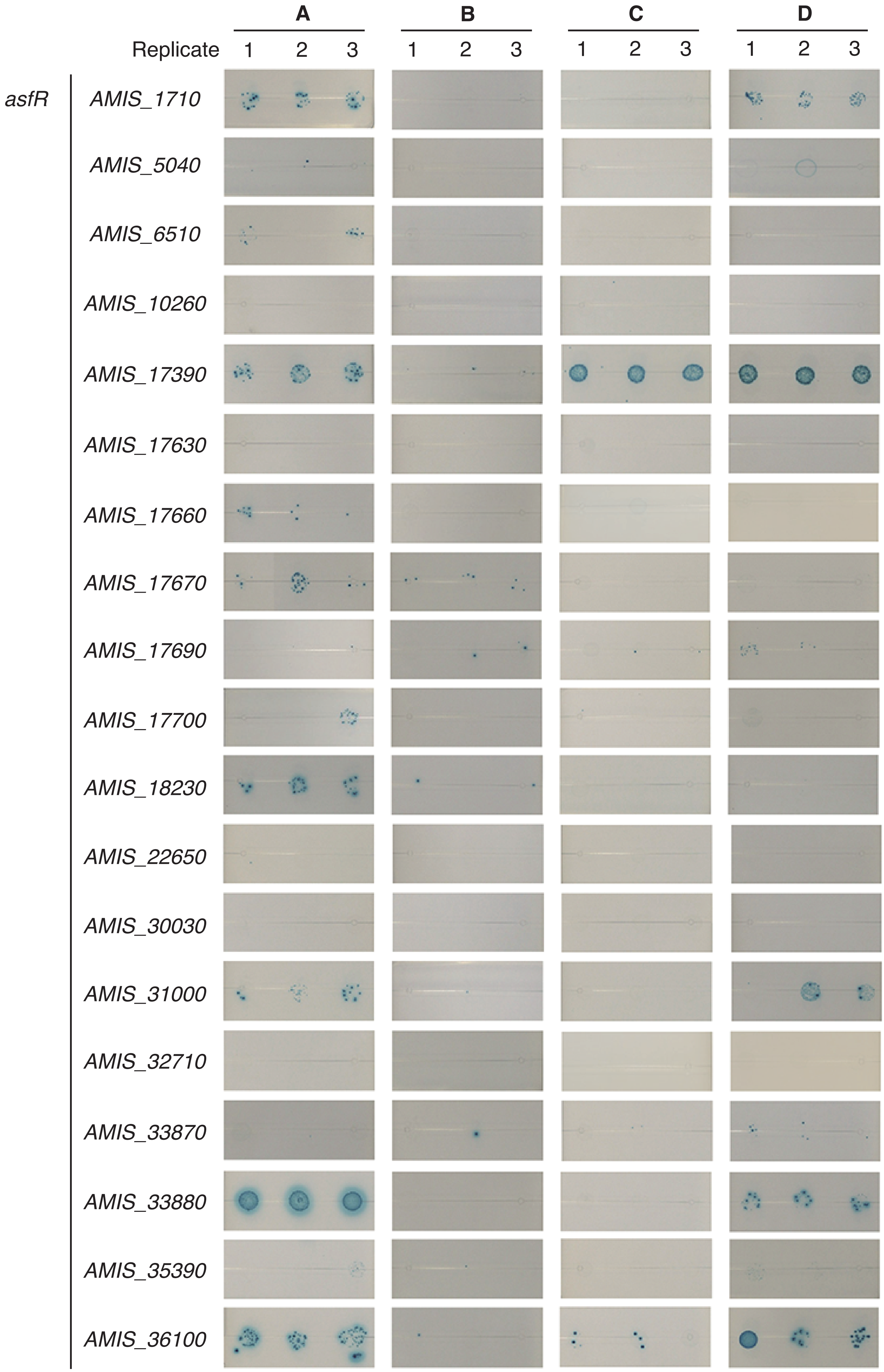

A
B
C
D
Replicate
1
2
3
asfR
AMIS_1710
AMIS_5040
AMIS_6510
AMIS_10260
AMIS_17390
AMIS_17630
AMIS_17660
AMIS_17670
AMIS_17690
AMIS_17700
AMIS_18230
AMIS_22650
AMIS_30030
AMIS_31000
AMIS_32710
AMIS_33870
AMIS_33880
AMIS_35390
AMIS_36100

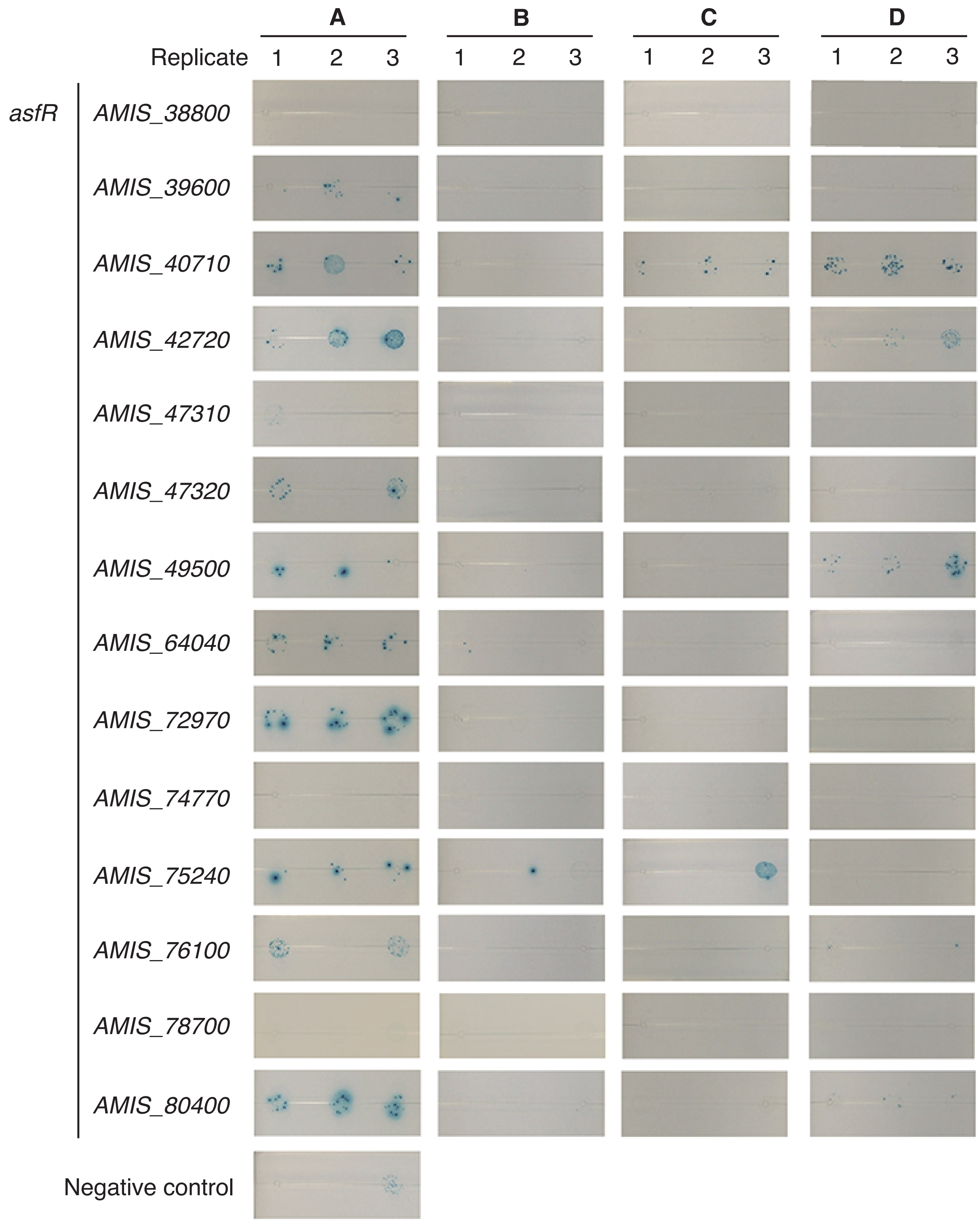

Fig. S5. Bacterial two-hybrid assay of AsfR and 33 HKs. *E. coli* BTH101 cells carrying the plasmids encoding protein fusions to the T18 or T25 domains were spotted onto M63 agar plates supplemented with X-Gal, IPTG, and maltose. The cells carrying the plasmids encoding the T18 or T25 domains were used as negative controls. The plates were incubated at 30°C for 4 days and photographed. The cells can grow and turn blue only when the T18 domain binds to the T25 domain through the interaction between two proteins fused individually to the T18 and T25 domains. *asfR* was cloned into pUT18C (A, D) or pUT18 (B, C), whereas HK genes were cloned into pKT25 (A, B) or pKNT25 (C, D).

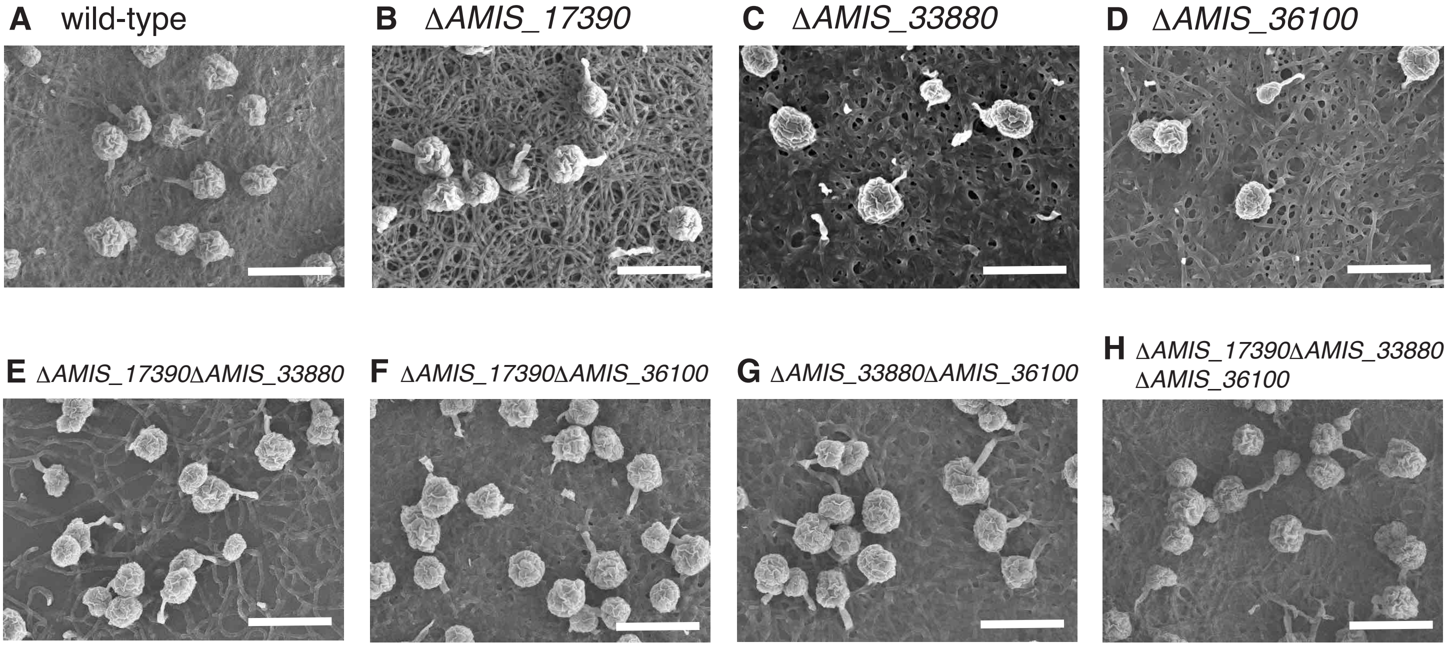

Fig. S6. SEM observation of sporangia and mycelia produced on HAT agar after 7 days of cultivation. (A) Wild-type strain. (B) Δ*AMIS_17390* strain. (C) Δ*AMIS_33880* strain. (D) Δ*AMIS_36100* strain. (E) Δ*AMIS_17390*Δ*AMIS_33880* strain. (F) Δ*AMIS_17390*Δ*AMIS_36100* strain. (G) Δ*AMIS_33880*Δ*AMIS_361000* strain. (H) Δ*AMIS_17390*Δ*AMIS_33880*Δ*AMIS_36100* strain. Scale bars, 10 μm. Panel A is the same image shown in Fig. 2A.

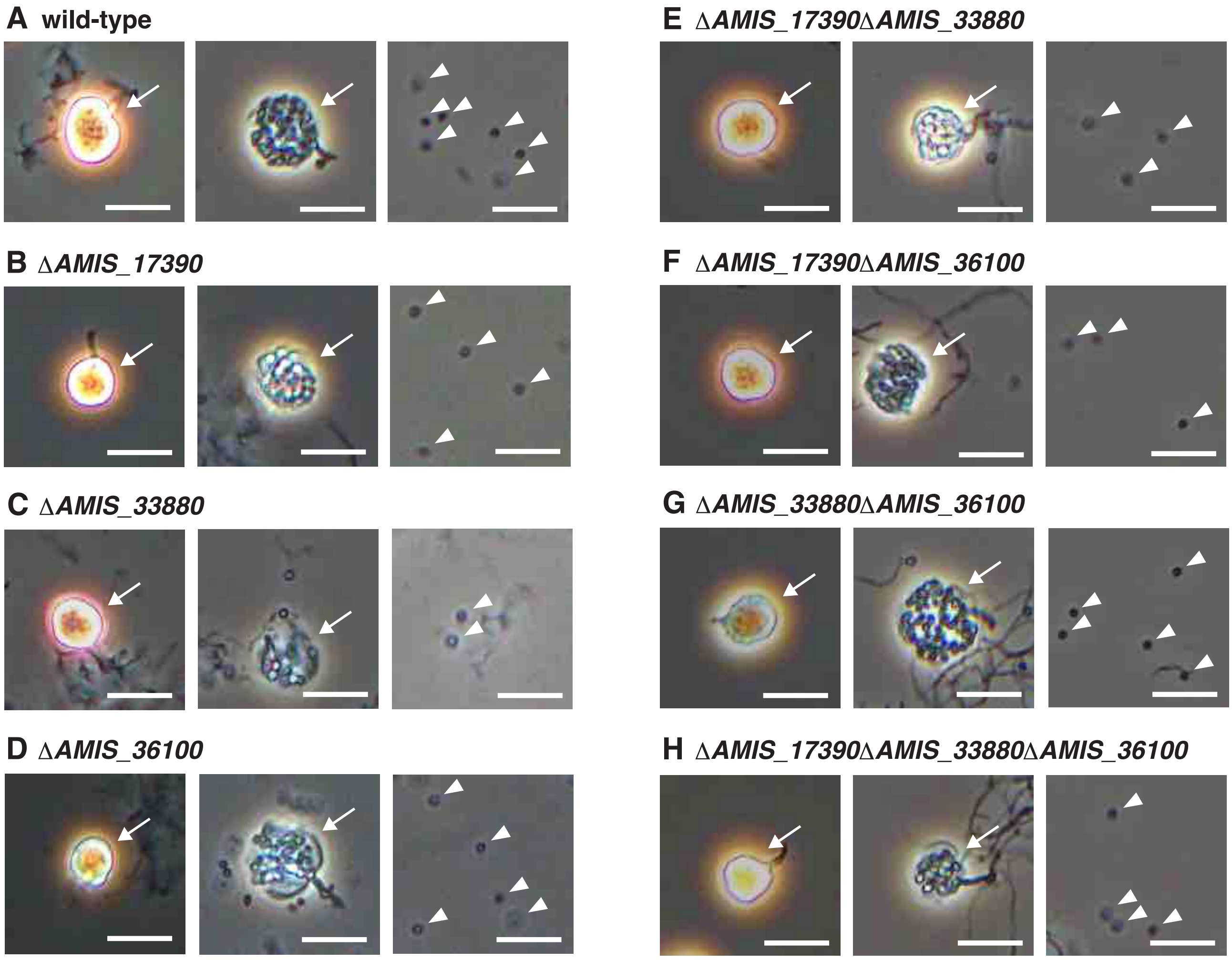

Fig. S7. Observation of sporangium dehiscence in the wild-type and deletion mutant strains of HK genes. Sporangia produced on HAT agar were harvested and suspended in 25 mM histidine solution to induce sporangium dehiscence. Micrographs of the wild-type (A), Δ*AMIS_17390* (B), Δ*AMIS_33880* (C), Δ*AMIS_36100* (D), Δ*AMIS_17390*Δ*AMIS_33880* (E), Δ*AMIS_17390*Δ*AMIS_36100* (F), Δ*AMIS_33880*Δ*AMIS_36100* (G), Δ*AMIS_17390*Δ*AMIS_33880*Δ*AMIS_36100* (H) strains are shown. Images in the left panels were obtained immediately after suspension. Images in the middle panels were obtained 15 min after suspension. Images in the right panels were obtained 30 min after suspension. Sporangia (including those whose membrane became transparent) and released spores are indicated by arrows and arrowheads, respectively. Scale bars, 10 μm.

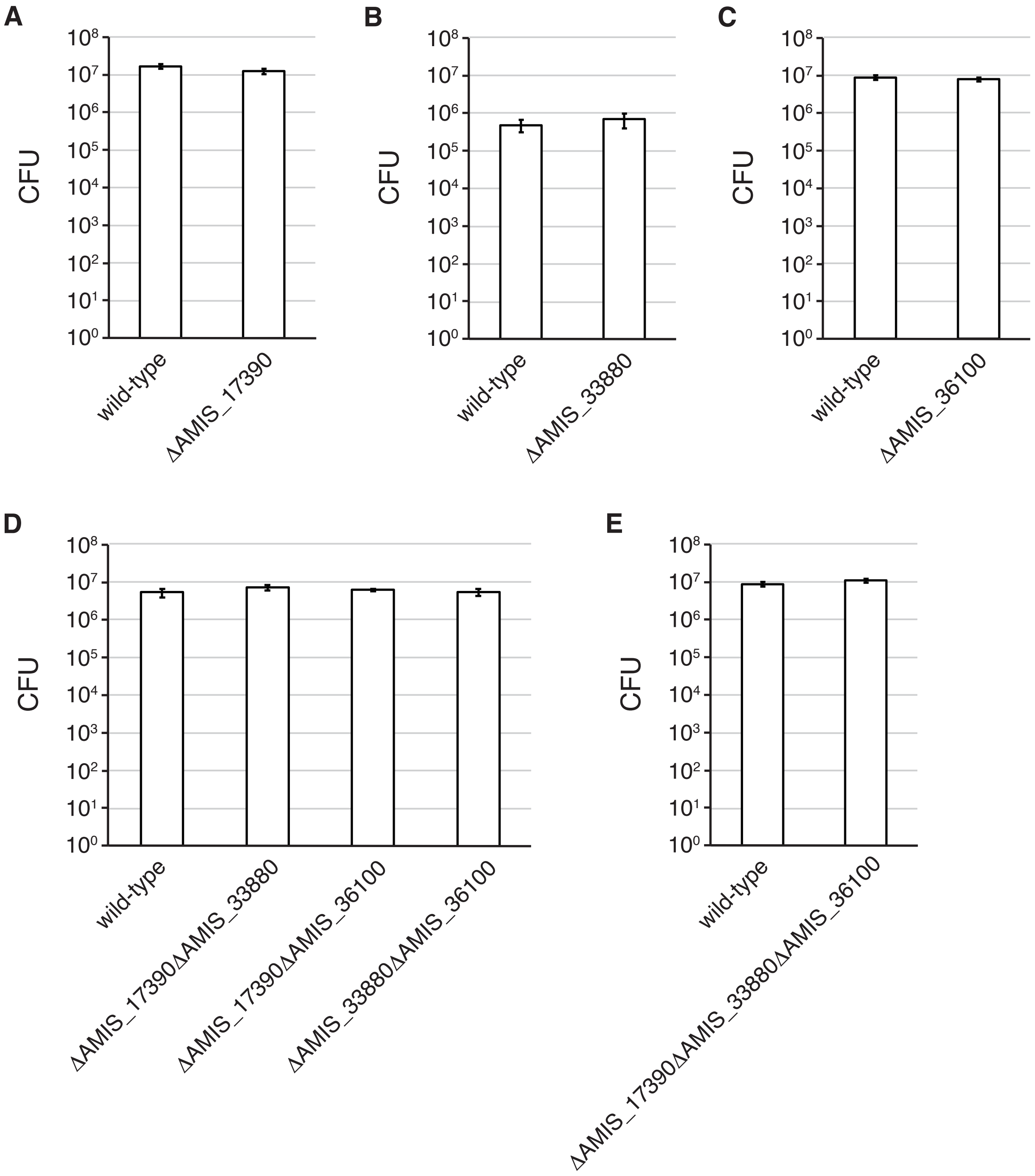

Fig. S8. Number of spores released from sporangia in the wild-type and deletion mutant strains of HK genes. Zoospores released from sporangia by pouring 25 mM NH_4HCO_3 solution were counted as CFU on YBNM agar. Data are mean values ± standard errors from three biological replicates. In A–E, the wild-type strain was used as a control.

Table S1. Primers used in this study

Primer name	Sequence (5' to 3')[a]	Restriction enzyme	Used for
76070-up-F	GGAATTCCCTCTTCGTCCAGGTGATTCCG	EcoRI	Gene disruption
76070-up-R	GCTCTAGAGTTCCTCACGCTATCGGCCG	XbaI	Gene disruption
76070-down-F	GCTCTAGAAAGACGGTCCTCGCGTAGAGG	XbaI	Gene disruption
76070-down-R	GCCAAGCTTCAGCTCCTCCTTGTCGAGACCC	HindIII	Gene disruption
AMIS_17390-UF2	CGGGATCCCCACGAGCACATCGATCTCA	BamHI	Gene disruption
AMIS_17390-UR1	GCTCTAGACCTCGTCGATCGAGACGAGT	XbaI	Gene disruption
AMIS_17390-DF1	GCTCTAGACTCTACATCGTCCGCAGCCT	XbaI	Gene disruption
AMIS_17390-DR1	GCCAAGCTTAGTGTGCTGGTGATCGTGGT	HindIII	Gene disruption
AMIS_33880-UF1	GGAATTCGGACTGCTCGGAATGGTGTA	EcoRI	Gene disruption
AMIS_33880-UR2	GCTCTAGAATCGCGCACATCGAATCGGA	XbaI	Gene disruption
AMIS_33880-DF1	GCTCTAGAAGCTGCCTGCGCATCGTCGA	XbaI	Gene disruption
AMIS_33880-DR1	GCCAAGCTTGATCGACTGCTCCTGCTGTA	HindIII	Gene disruption
AMIS_36100-UF1	GGAATTCAGGTCAGCGAGGTCCACGAA	EcoRI	Gene disruption
AMIS_36100-UR1	GCTCTAGACTGACCAGCTCGGTCTTGGA	XbaI	Gene disruption
AMIS_36100-DF1	GCTCTAGAAACGCCATGGCGCTGATGCT	XbaI	Gene disruption
AMIS_36100-DR1	GCCAAGCTTGTCCCGGAGTTGCGGATCTT	HindIII	Gene disruption
76070-int-F	CTGGTGGACAACTCGGTGTC		Colony PCR
76070-int-R	AGCGATGGCTCGCCACGGTA		Colony PCR
AMIS_17390-CF1	ACAGAGCAGGCCACGGACCA		Colony PCR
AMIS_17390-CR1	GAATCCGGCGGCTGCCGTGT		Colony PCR
AMIS_33880-CF1	CTGGCCGCGTTGCACGAGTA		Colony PCR
AMIS_33880-CR1	GAGAGCGAGTGGGCAGCGTA		Colony PCR
AMIS_36100-CF3	CTCCAAGACCGAGCTGGTCA		Colony PCR
AMIS_36100-CR3	AGCATCAGCGCCATGGCGTT		Colony PCR
76070-SB-F	CTGGTGGACAACTCGGTGTC		Southern blotting
76070-SB-R	GCTCTAGAGTTCCTCACGCTATCGGCCG		Southern blotting
76070_comp-F	GGAATTCCATCCCGTCGACGCGTCCAG	EcoRI	Gene complementation
76070_comp-R	GCTCTAGACTACGCGAGGACCGTCTTC	XbaI	Gene complementation
76070_D72N-F	CCTGGTCACCAACCTCACCCT		Gene complementation
76070_D72N-R	AGGGTGAGGTTGGTGACCAGG		Gene complementation
AMIS_76070-F1	GCTCTAGAGATGAGGAACGACCCCAGCCCC	XbaI	Bacterial two-bybrid assay
AMIS_76070-R1	TCCCCCGGGGCGCGAGGACCGTCTTCAGTG	SmaI	Bacterial two-bybrid assay
AMIS_1710-F1	GCTCTAGAGATGACCGGACCGACCGGCGA	XbaI	Bacterial two-bybrid assay
AMIS_1710-R1	TCCCCCGGGGTGACGTCGCGGGCAGGCGCA	SmaI	Bacterial two-bybrid assay
AMIS_5040-F1	GCTCTAGAGATGAGCTACCGGATCTCACTGGC	XbaI	Bacterial two-bybrid assay
AMIS_5040-R1	GGGGTACCCGCAGCGCCACGACCGCACCCGAA	KpnI	Bacterial two-bybrid assay
AMIS_6510-F2	AAAACTGCAGGATGAGCACGGGATCCTCGGCC	PstI	Bacterial two-bybrid assay
AMIS_6510-R1	GCTCTAGAGTCTGTTCTTTCTCCTTAGGAGCATG	XbaI	Bacterial two-bybrid assay
AMIS_10260-F1	GCTCTAGAGATGAGAAGCCGCAACTGGTC	XbaI	Bacterial two-bybrid assay
AMIS_10260-R1	GGGGTACCCGTGCGTCCTTCTCCAGCCTGA	KpnI	Bacterial two-bybrid assay
AMIS17390-F1	GCTCTAGAGGTGGCCCAGAACGCGATCA	XbaI	Bacterial two-bybrid assay
AMIS17390-R2	GGAATTCGACGCCGGCCCGGCGGGGTCGG	EcoRI	Bacterial two-bybrid assay
AMIS_17630-F1	GCTCTAGAGATGGTGGCCGTCATCGGTTG	XbaI	Bacterial two-bybrid assay
AMIS_17630-R1	GGGGTACCCGCGCGGAGGCCACCAGGCTCGT	Kpn	Bacterial two-bybrid assay
AMIS_17660-BT-F1	GCTCTAGAGATGTCCGTTCCCGGCACGG	XbaI	Bacterial two-bybrid assay
AMIS_17660-BT-R2	GGAATTCGAACGGGTGGGGGTCCGCGA	EcoRI	Bacterial two-bybrid assay
AMIS_17670-F1	GCTCTAGAGATGGCCGCTACCAAGGACCC	XbaI	Bacterial two-bybrid assay
AMIS_17670-R1	GGGGTACCCGGAAGGCGCTCTCCAGCGTCA	KpnI	Bacterial two-bybrid assay

AMIS_17690-F1	GCTCTAGAGATGAGCCAAGATCCGGACGA	XbaI	Bacterial two-bybrid assay
AMIS_17690-R1	GGGGTACCCGGCGGGCGCCGGGCAGGCGGAT	KpnI	Bacterial two-bybrid assay
AMIS_17700-F1	GCTCTAGAGATGACCTATCGCAGGTCGCT	XbaI	Bacterial two-bybrid assay
AMIS_17700-R1	GGGGTACCCGTCGGCGCGGTTCCCTCCACG	KpnI	Bacterial two-bybrid assay
AMIS_18230-F1	GCTCTAGAGATGCTCGATCTCGAAGAGGC	XbaI	Bacterial two-bybrid assay
AMIS_18230-R1	TCCCCCGGGGTCGGATACTGGCGGCACGGT	SmaI	Bacterial two-bybrid assay
AMIS_22650-F1	GCTCTAGAGATGGTCCCCAGTGTCGTGGC	XbaI	Bacterial two-bybrid assay
AMIS_22650-R2	GGAATTCGATGCGTTCCTGCCTTGCTCAG	EcoRI	Bacterial two-bybrid assay
AMIS_31000-F1	GCTCTAGAGATGGTCGTGGGGCAGGGCAG	XbaI	Bacterial two-bybrid assay
AMIS_31000-R1	GGGGTACCCGCCTCCCGGTTGGCGGCGCGG	KpnI	Bacterial two-bybrid assay
AMIS_32710-BT-F1	GCTCTAGAGATGGGGCACAGGAGCGGTG	XbaI	Bacterial two-bybrid assay
AMIS_32710-BT-R1	GGAATTCGAGGGGCGGCGGGAACCACG	EcoRI	Bacterial two-bybrid assay
AMIS_33870-F1	GCTCTAGAGATGCAGGGGGCGGACCGGGA	XbaI	Bacterial two-bybrid assay
AMIS_33870-R1	GGGGTACCCGCGCCGTATCTGACCGGGCCG	KpnI	Bacterial two-bybrid assay
AMIS_33880-F1	GCTCTAGAGATGGACGTCGAGCGCGAGCG	XbaI	Bacterial two-bybrid assay
AMIS_33880-R1	TCCCCCGGGGATCGGGGAGTGAGAAGATCA	SmaI	Bacterial two-bybrid assay
AMIS35390-F1	GCTCTAGAGATGGCCGTGGAGGACGCCCC	XbaI	Bacterial two-bybrid assay
AMIS35390-R1	GGGGTACCCGCTGGCGGGTGGCCGAGAACT	KpnI	Bacterial two-bybrid assay
AMIS36100-F1	GCTCTAGAGATGTTCGCATTGTGGCCGTT	XbaI	Bacterial two-bybrid assay
AMIS36100-R1	GGGGTACCCGCCGGGGGAGGGTGGCGCACA	KpnI	Bacterial two-bybrid assay
AMIS38800-F1	GCTCTAGAGATGGTCGCCGGCCTGCTCGC	XbaI	Bacterial two-bybrid assay
AMIS38800-R1	GGGGTACCCGTGGAGTCTGTTGTAGGGGAAA	KpnI	Bacterial two-bybrid assay
AMIS_39600-F1	GCTCTAGAGATGGAGGTCAACGACGCCTA	XbaI	Bacterial two-bybrid assay
AMIS_39600-R1	TCCCCCGGGGGCCTCGCGGCGGCGTGCCGG	SmaI	Bacterial two-bybrid assay
AMIS_40710-F1	GCTCTAGAGATGACCCGCTCGGCGGCCGA	XbaI	Bacterial two-bybrid assay
AMIS_40710-R1	GGGGTACCCGGGACGGCCAGCGCGGCAGCT	KpnI	Bacterial two-bybrid assay
AMIS_42720-F1	GCTCTAGAGATGGTGACGGAGAACGACGC	XbaI	Bacterial two-bybrid assay
AMIS_42720-R1	GGGGTACCCGGGCCTTCGGCAGGCGCACCC	KpnI	Bacterial two-bybrid assay
AMIS_47310-F1	GCTCTAGAGATGAGTTACCGGATCTCACT	XbaI	Bacterial two-bybrid assay
AMIS_47310-R1	GGGGTACCCGGGCCGAGGTCATCAGCTGGT	KpnI	Bacterial two-bybrid assay
AMIS_47320-F1	GCTCTAGAGATGGCCGTGAACGAGGACTC	XbaI	Bacterial two-bybrid assay
AMIS_47320-R1	GGGGTACCCGGGGGTGGGTGCTCGGCAGGT	KpnI	Bacterial two-bybrid assay
AMIS49500-F1	GCTCTAGAGATGAGCGTGCAGCGACGTCT	XbaI	Bacterial two-bybrid assay
AMIS49500-R1	GGGGTACCCGGCCCACCGGGACCGACTCCG	KpnI	Bacterial two-bybrid assay
AMIS_64040-F1	GCTCTAGAGATGACCGCACCTCACGGGGG	XbaI	Bacterial two-bybrid assay
AMIS_64040-R1	GGGGTACCCGGGTGACTTCCAGGGGGAGGC	KpnI	Bacterial two-bybrid assay
AMIS72970-F1	GCTCTAGAGATGTCCACCCTGCGTGACCT	XbaI	Bacterial two-bybrid assay
AMIS72970-R1	GGGGTACCCGGCGCTTCGCCAGCGGCACCA	KpnI	Bacterial two-bybrid assay
AMIS_74770-F1	GCTCTAGAGATGAGCAAGCGCCCCAAGGC	XbaI	Bacterial two-bybrid assay
AMIS_74770-R2	GGAATTCGATGCCTCATGCTCCTTGCCAG	EcoRI	Bacterial two-bybrid assay
AMIS_75240-F1	GCTCTAGAGATGAAAGCTCCACTGCCCGA	XbaI	Bacterial two-bybrid assay
AMIS_75240-R2	GGAATTCGAGGCGATCGCCAGGTCCCGGA	EcoRI	Bacterial two-bybrid assay
AMIS76100-F1	GCTCTAGAGATGGGCAGGCTGTCCCGGCT	XbaI	Bacterial two-bybrid assay
AMIS76100-R2	GGAATTCGACGCCGGTGCTCGCTCCAGGG	EcoRI	Bacterial two-bybrid assay
AMIS_78700-BT-F1	GCTCTAGAGGTGCTCTTCGGTAGGTTCCG	XbaI	Bacterial two-bybrid assay
AMIS_78700-BT-R1s	GGAATTCTTATCGCGGTTGTCCCTCGTGTT	EcoRI	Bacterial two-bybrid assay
AMIS80400-F1	GCTCTAGAGATGCCGGACCGTCCCGACTA	XbaI	Bacterial two-bybrid assay
AMIS80400-R1	GGGGTACCCGGGCGCGGGGCAGGCGAACCT	KpnI	Bacterial two-bybrid assay

[a] The recognition sequences for restriction enzymes are underlined.

Table S2. Putative sensor HK genes on the *A . missouriensis* genome[a]

Gene ID	Length (aa)	Conserved domain[b]	Response regulator gene	Length (aa)	Conserved domain[b]
AMIS_1710	450	Sensor histidine kinase regulatory (IPR050736)			
AMIS_1810	1,467	Hisitidine kinase domain (IPR005467); Signal transduction response regulator, receiver domain (IPR001789); GAF domain (IPR003018); HAMP domain (IPR003660)	AMIS_1820	182	Signal transduction response regulator, receiver domain (IPR001789)
AMIS_4280	440	Sensor histidine kinase two-component system (IPR050482); Putative sensor domain (IPR025828)	AMIS_4290	214	Signal transduction response regulator, receiver domain (IPR001789); Transcription regulator LuxR, C-terminal (IPR000792)
AMIS_5040	697	Hisitidine kinase domain (IPR005467); PAS fold-4 (IPR013656); MASE1 (IPR007895)			
AMIS_5120	520	Two-component system sensor histidine kinase (IPR050428)	AMIS_5130	274	Signal transduction response regulator, receiver domain (IPR001789); OmpR/PhoB-type DNA-binding domain (IPR001867)
AMIS_5190	533	Histidine kinase domain (IPR005467); HAMP domain (IPR003660); CHASE3 (IPR007891)	AMIS_5200	150	Signal transduction response regulator, receiver domain (IPR001789)
AMIS_6510	992	Two-component sensor histidine kinase (IPR050980)			
AMIS_7030	409	Sensor histidine kinase two-component system (IPR050482)	AMIS_7040	222	Signal transduction response regulator, receiver domain (IPR001789); Transcription regulator LuxR, C-terminal (IPR000792)
AMIS_8740	526	MtrAB system histidine kinase MtrB (IPR047669)	AMIS_8730	232	MtrAB system response regulator MtrA (IPR047671)
AMIS_10260	875	Two-component sensor histidine kinase (IPR050980)			
AMIS_11500	500	Hisitidine kinase domain (IPR005467); Signal transduction response regulator, receiver domain (IPR001789); PAS fold (IPR013767)			
AMIS_12140	405	Sensor histidine kinase two-component system (IPR050482)	AMIS_12150	245	Signal transduction response regulator, receiver domain (IPR001789); Transcription regulator LuxR, C-terminal (IPR000792)
AMIS_14390	537	Sensor histidine kinase two-component system (IPR050482)	AMIS_14380	221	Signal transduction response regulator, receiver domain (IPR001789); Transcription regulator LuxR, C-terminal (IPR000792)
AMIS_16680	391	Sensor histidine kinase two-component system (IPR050482)	AMIS_16670	215	Signal transduction response regulator, receiver domain (IPR001789); Transcription regulator LuxR, C-terminal (IPR000792)
AMIS_17030	434	Sensor histidine kinase two-component system (IPR050482)	AMIS_17040	225	Signal transduction response regulator, receiver domain (IPR001789); Transcription regulator LuxR, C-terminal (IPR000792)
AMIS_17340	577	Hisitidine kinase domain (IPR005467); PAS domain (IPR000014); Signal transduction response regulator, receiver domain (IPR001789)			
AMIS_17390	924	Hisitidine kinase domain (IPR005467); PAS domain (IPR000014); GAF domain (IPR003018)			
AMIS_17480	832	Sensor histidine kinase KdpD (IPR052023)	AMIS_17500	221	Signal transduction response regulator, receiver domain (IPR001789); OmpR/PhoB-type DNA-binding domain (IPR001867)
AMIS_17490	475	Sensor histidine kinase KdpD (IPR052023)			
AMIS_17630	705	Hisitidine kinase domain (IPR005467); PAS domain (IPR000014); CHASE domain (IPR006189)			
AMIS_17660	673	Hisitidine kinase domain (IPR005467); PAS domain (IPR000014)			
AMIS_17670	780	Hisitidine kinase domain (IPR005467); PAS domain (IPR000014); GAF domain (IPR003018)			
AMIS_17690	397	Hisitidine kinase domain (IPR005467); GAF domain (IPR003018)			
AMIS_17700	704	Hisitidine kinase domain (IPR005467); PAS domain (IPR000014); MASE1 (IPR007895)			
AMIS_18230	424	Hisitidine kinase domain (IPR005467); GAF domain (IPR003018)			
AMIS_18610	669	Hisitidine kinase domain (IPR005467); Signal transduction response regulator, receiver domain (IPR001789); PAS domain (IPR000014)			
AMIS_18620	1,072	Hisitidine kinase domain (IPR005467); Signal transduction response regulator, receiver domain (IPR001789); Phosphotransfer (Hpt) domain (IPR008207); PAS domain (IPR000014)			
AMIS_18930	635	Hisitidine kinase domain (IPR005467); Signal transduction response regulator, receiver domain (IPR001789); PAS domain (IPR000014)			
AMIS_22050	518	Sensor histidine kinase two-component system (IPR050482)	AMIS_22060	217	Signal transduction response regulator, receiver domain (IPR001789); Transcription regulator LuxR, C-terminal (IPR000792)
AMIS_22650	778	Sensor histidine kinase two-component system (IPR050482)			
AMIS_23390	248	Sensor histidine kinase two-component system (IPR050482)	AMIS_23380	219	Signal transduction response regulator, receiver domain (IPR001789); Transcription regulator LuxR, C-terminal (IPR000792)
AMIS_23790	381	Sensor histidine kinase two-component system (IPR050482)	AMIS_23780	204	Signal transduction response regulator, receiver domain (IPR001789); Transcription regulator LuxR, C-terminal (IPR000792)
AMIS_24020	474	Sensor histidine kinase two-component system (IPR050482)	AMIS_24030	227	Signal transduction response regulator, receiver domain (IPR001789); Transcription regulator LuxR, C-terminal (IPR000792)
AMIS_25030	778	Hisitidine kinase domain (IPR005467); Signal transduction response regulator, receiver domain (IPR001789); HAMP domain (IPR003660); CHASE4 (IPR007892)			
AMIS_25640	652	Hisitidine kinase domain (IPR005467); Signal transduction response regulator, receiver domain (IPR001789); PAS domain (IPR000014)			
AMIS_25700	675	Sensor histidine kinase two-component system (IPR050482)	AMIS_25710	120	Signal transduction response regulator, receiver domain (IPR001789)
AMIS_26080	583	Hisitidine kinase domain (IPR005467); HAMP domain (IPR003660)	AMIS_26090	227	Signal transduction response regulator, receiver domain (IPR001789); OmpR/PhoB-type DNA-binding domain (IPR001867)
AMIS_26160	586	Hisitidine kinase domain (IPR005467); HAMP domain (IPR003660)	AMIS_26150	226	Signal transduction response regulator, receiver domain (IPR001789); OmpR/PhoB-type DNA-binding domain (IPR001867)
AMIS_26210	389	Sensor histidine kinase two-component system (IPR050482)	AMIS_26200	219	Signal transduction response regulator, receiver domain (IPR001789); Transcription regulator LuxR, C-terminal (IPR000792)
AMIS_26530	417	Sensor histidine kinase two-component system (IPR050482)	AMIS_26520	225	Signal transduction response regulator, receiver domain (IPR001789); Transcription regulator LuxR, C-terminal (IPR000792)
AMIS_26850	401	Sensor histidine kinase two-component system (IPR050482)	AMIS_26860	225	Signal transduction response regulator, receiver domain (IPR001789); Transcription regulator LuxR, C-terminal (IPR000792)
AMIS_27440	398	Sensor histidine kinase two-component system (IPR050482)	AMIS_27430	218	Signal transduction response regulator, receiver domain (IPR001789); Transcription regulator LuxR, C-terminal (IPR000792)
AMIS_29050	1,503	Hisitidine kinase domain (IPR005467); Signal transduction response regulator, receiver domain (IPR001789); GAF domain (IPR003018); HAMP domain (IPR003660)			
AMIS_29120	436	Sensor histidine kinase two-component system (IPR050482)	AMIS_29110	214	Signal transduction response regulator, receiver domain (IPR001789); Transcription regulator LuxR, C-terminal (IPR000792)
AMIS_29360	415	Sensor histidine kinase two-component system (IPR050482)	AMIS_29370	219	Signal transduction response regulator, receiver domain (IPR001789); Transcription regulator LuxR, C-terminal (IPR000792)
AMIS_29980	339	Hisitidine kinase domain (IPR005467)	AMIS_29970	231	Signal transduction response regulator, receiver domain (IPR001789); OmpR/PhoB-type DNA-binding domain (IPR001867)
AMIS_30030	447	Two-component system sensor histidine kinase (IPR050428)			
AMIS_30320	450	Two-component system sensor histidine kinase (IPR050428)	AMIS_30330	227	Signal transduction response regulator, receiver domain (IPR001789); OmpR/PhoB-type DNA-binding domain (IPR001867)
AMIS_30420	374	Sensor histidine kinase two-component system (IPR050482)	AMIS_30430	223	Signal transduction response regulator, receiver domain (IPR001789); Transcription regulator LuxR, C-terminal (IPR000792)
AMIS_30890	1,810	Hybrid signal transduction histidine kinase (IPR053159); PAS domain (IPR000014); GAF domain (IPR003018)			
AMIS_30990	1,065	Hisitidine kinase domain (IPR005467); Signal transduction response regulator, receiver domain (IPR001789); PAS domain (IPR000014)			
AMIS_31000	279	Hisitidine kinase domain (IPR005467)			
AMIS_31010	653	Hisitidine kinase domain (IPR005467); Signal transduction response regulator, receiver domain (IPR001789); PAS domain (IPR000014)			
AMIS_31910	363	Sensor histidine kinase two-component system (IPR050482)	AMIS_31900	218	Signal transduction response regulator, receiver domain (IPR001789); OmpR/PhoB-type DNA-binding domain (IPR001867)
AMIS_32710	800	Two-component sensor histidine kinase (IPR050980)			
AMIS_33180	777	Histidine kinase domain (IPR005467); Signal transduction response regulator, receiver domain (IPR001789); PAS domain (IPR000014)			
AMIS_33620	409	Sensor histidine kinase two-component system (IPR050482)	AMIS_33630	214	Signal transduction response regulator, receiver domain (IPR001789); Transcription regulator LuxR, C-terminal (IPR000792)
AMIS_33870	371	Signal transduction histidine kinase (IPR003661); GAF domain (IPR003018); PAS domain superfamily (IPR035965)			
AMIS_33880	539	Histidine kinase domain (IPR005467); PAS domain (IPR000014); GAF-like domain superfamily (IPR029016)			
AMIS_34240	415	Sensor histidine kinase two-component system (IPR050482)	AMIS_34230	213	Signal transduction response regulator, receiver domain (IPR001789); Transcription regulator LuxR, C-terminal (IPR000792)
AMIS_34800	385	Sensor histidine kinase two-component system (IPR050482)	AMIS_34790	223	Signal transduction response regulator, receiver domain (IPR001789); Transcription regulator LuxR, C-terminal (IPR000792)
AMIS_35390	532	Histidine kinase domain (IPR005467); Membrane-associated sensor domain (IPR033424)			
AMIS_35530	612	Histidine kinase domain (IPR005467); Signal transduction response regulator, receiver domain (IPR001789)			
AMIS_35880	389	Sensor histidine kinase two-component system (IPR050482)	AMIS_35870	225	Signal transduction response regulator, receiver domain (IPR001789); Transcription regulator LuxR, C-terminal (IPR000792)
AMIS_36070	746	Histidine kinase domain (IPR005467); Signal transduction response regulator, receiver domain (IPR001789); Phosphotransfer (Hpt) domain (IPR008207); HAMP domain (IPR003660)	AMIS_36080	124	Signal transduction response regulator, receiver domain (IPR001789)
AMIS_36100	254	Signal transduction histidine kinase, dimerization/phosphoacceptor domain (IPR003661)			
AMIS_36880	771	Histidine kinase domain (IPR005467); Signal transduction response regulator, receiver domain (IPR001789); PAS domain (IPR000014)			
AMIS_37400	558	Sensor histidine kinase two-component system (IPR050482)	AMIS_37410	213	Signal transduction response regulator, receiver domain (IPR001789); Transcription regulator LuxR, C-terminal (IPR000792)
AMIS_37440	394	Hisitidine kinase domain (IPR005467); GAF domain (IPR003018)	AMIS_37450	388	Signal transduction response regulator, receiver domain (IPR001789)

AMIS_37680 (*rsdK*)	706	Hisitidine kinase domain (IPR005467); GAF domain (IPR003018); PAS domain (IPR000014)	AMIS_37670 (*rsdR*)	151	Signal transduction response regulator, receiver domain (IPR001789)
AMIS_38800	362	Histidine kinase domain (IPR005467)			
AMIS_39220	706	Sensor histidine kinase two-component system (IPR050482)	AMIS_39230	207	Signal transduction response regulator, receiver domain (IPR001789); Transcription regulator LuxR, C-terminal (IPR000792)
AMIS_39240	1,233	Hisitidine kinase domain (IPR005467); Signal transduction response regulator, receiver domain (IPR001789); PPM-type phosphatase-like domain (IPR001932); GAF-like domain superfamily (IPR029016); PAS fold-4 (IPR013656)			
AMIS_39600	654	Histidine kinase domain (IPR005467); PAS fold-4 (IPR013656)			
AMIS_40320	417	Histidine kinase domain (IPR005467); HAMP domain (IPR003660)	AMIS_40330	226	Signal transduction response regulator, receiver domain (IPR001789); OmpR/PhoB-type DNA-binding domain (IPR001867)
AMIS_40710	510	Histidine kinase domain (IPR005467); PAS fold-4 (IPR013656); GAF domain (IPR003018)			
AMIS_41290	372	Sensor histidine kinase two-component system (IPR050482)	AMIS_41300	203	Signal transduction response regulator, receiver domain (IPR001789); Transcription regulator LuxR, C-terminal (IPR000792)
AMIS_41440	356	Sensor histidine kinase two-component system (IPR050482)	AMIS_41450	222	Signal transduction response regulator, receiver domain (IPR001789); Transcription regulator LuxR, C-terminal (IPR000792)
AMIS_42290	395	Sensor histidine kinase two-component system (IPR050482)	AMIS_42300	220	Signal transduction response regulator, receiver domain (IPR001789); Transcription regulator LuxR, C-terminal (IPR000792)
AMIS_42720	699	Histidine kinase domain (IPR005467); PAS domain (IPR000014); GAF domain (IPR003018)			
AMIS_42890	363	Sensor histidine kinase two-component system (IPR050482)	AMIS_42900	222	Signal transduction response regulator, receiver domain (IPR001789); Transcription regulator LuxR, C-terminal (IPR000792)
AMIS_44800	403	Sensor histidine kinase two-component system (IPR050482)	AMIS_44810	214	Signal transduction response regulator, receiver domain (IPR001789); Transcription regulator LuxR, C-terminal (IPR000792)
AMIS_46400	390	Sensor histidine kinase two-component system (IPR050482)	AMIS_46390	201	Signal transduction response regulator, receiver domain (IPR001789); Transcription regulator LuxR, C-terminal (IPR000792)
AMIS_46790	1,360	Histidine kinase domain (IPR005467); PPM-type phosphatase-like domain (IPR001932); Signal transduction response regulator, receiver domain (IPR001789); PAS domain (IPR000014); GAF-like domain superfamily (IPR029016)			
AMIS_47080	378	Sensor histidine kinase two-component system (IPR050482)	AMIS_47070	201	Signal transduction response regulator, receiver domain (IPR001789); Transcription regulator LuxR, C-terminal (IPR000792)
AMIS_47310	689	Histidine kinase domain (IPR005467); PAS domain (IPR000014)			
AMIS_47320	661	Histidine kinase domain (IPR005467); PAS domain (IPR000014); GAF domain (IPR003018)			
AMIS_47360	203	Histidine kinase domain (IPR005467)	AMIS_47350	214	Signal transduction response regulator, receiver domain (IPR001789); Transcription regulator LuxR, C-terminal (IPR000792)
AMIS_47720	296	Sensor histidine kinase two-component system (IPR050482)	AMIS_47710	221	Signal transduction response regulator, receiver domain (IPR001789); Transcription regulator LuxR, C-terminal (IPR000792)
AMIS_48730	488	Two-component system sensor histidine kinase (IPR050428)	AMIS_48740	239	Signal transduction response regulator, receiver domain (IPR001789); OmpR/PhoB-type DNA-binding domain (IPR001867)
AMIS_49010	403	Sensor histidine kinase two-component system (IPR050482)	AMIS_49000	210	Signal transduction response regulator, receiver domain (IPR001789); Transcription regulator LuxR, C-terminal (IPR000792)
AMIS_49320	432	Sensor histidine kinase two-component system (IPR050482)	AMIS_49310	217	Signal transduction response regulator, receiver domain (IPR001789); Transcription regulator LuxR, C-terminal (IPR000792)
AMIS_49500	646	Histidine kinase domain (IPR005467); HAMP domain (IPR003660); CHASE3 (IPR007891)			
AMIS_49820	660	Sensor histidine kinase two-component system (IPR050482)	AMIS_49810	220	Signal transduction response regulator, receiver domain (IPR001789); Transcription regulator LuxR, C-terminal (IPR000792)
AMIS_49930	451	Two-component system sensor histidine kinase (IPR050428)	AMIS_49940	246	Signal transduction response regulator, receiver domain (IPR001789); OmpR/PhoB-type DNA-binding domain (IPR001867)
AMIS_51350	370	Two-component system sensor histidine kinase (IPR050428)	AMIS_51360	216	Signal transduction response regulator, receiver domain (IPR001789); OmpR/PhoB-type DNA-binding domain (IPR001867)
AMIS_51700	590	Two-component system sensor histidine kinase (IPR050428)	AMIS_51710	234	Signal transduction response regulator, receiver domain (IPR001789); OmpR/PhoB-type DNA-binding domain (IPR001867)
AMIS_51750	362	Two-component system sensor histidine kinase (IPR050428)	AMIS_51740	221	Signal transduction response regulator, receiver domain (IPR001789); OmpR/PhoB-type DNA-binding domain (IPR001867)
AMIS_52460	651	Histidine kinase domain (IPR005467); Signal transduction response regulator, receiver domain (IPR001789)	AMIS_52450 (*cheB1*)	355	Signal transduction response regulator, receiver domain (IPR001789); Signal transduction response regulator, chemotaxis, protein-glutamate methylesterase (IPR000673)
AMIS_52500	387	Sensor histidine kinase two-component system (IPR050482)	AMIS_52510	234	Signal transduction response regulator, receiver domain (IPR001789); Transcription regulator LuxR, C-terminal (IPR000792)
AMIS_52850	654	Sensor histidine kinase two-component system (IPR050482)	AMIS_52860	214	Signal transduction response regulator, receiver domain (IPR001789); Transcription regulator LuxR, C-terminal (IPR000792)
AMIS_52990	468	Two-component system sensor histidine kinase (IPR050428)	AMIS_53000	235	Signal transduction response regulator, receiver domain (IPR001789); OmpR/PhoB-type DNA-binding domain (IPR001867)
AMIS_53200	447	Sensor histidine kinase two-component system (IPR050482)	AMIS_53210	216	Signal transduction response regulator, receiver domain (IPR001789); Transcription regulator LuxR, C-terminal (IPR000792)
AMIS_53840	383	Sensor histidine kinase two-component system (IPR050482)	AMIS_53850	230	Signal transduction response regulator, receiver domain (IPR001789); Transcription regulator LuxR, C-terminal (IPR000792)
AMIS_53880	390	Sensor histidine kinase two-component system (IPR050482)	AMIS_53890	229	Signal transduction response regulator, receiver domain (IPR001789); Transcription regulator LuxR, C-terminal (IPR000792)
AMIS_54460	744	Sensor histidine kinase regulatory (IPR050736); CHASE domain (IPR006189)	AMIS_54450	160	Signal transduction response regulator, receiver domain (IPR001789)
AMIS_55270	396	Sensor histidine kinase two-component system (IPR050482)	AMIS_55260	213	Signal transduction response regulator, receiver domain (IPR001789); Transcription regulator LuxR, C-terminal (IPR000792)
AMIS_58500	509	Two-component system sensor histidine kinase (IPR050428)	AMIS_58490	231	Signal transduction response regulator, receiver domain (IPR001789); Winged helix DNA-binding domain superfamily (IPR036390)
AMIS_62990	472	Two-component sensor histidine kinase (IPR050980)	AMIS_62980	229	Signal transduction response regulator, receiver domain (IPR001789); OmpR/PhoB-type DNA-binding domain (IPR001867)
AMIS_63420	425	Sensor histidine kinase two-component system (IPR050482)	AMIS_63410	208	Signal transduction response regulator, receiver domain (IPR001789); Transcription regulator LuxR, C-terminal (IPR000792)
AMIS_64040	708	Histidine kinase domain (IPR005467); GAF domain (IPR003018); PAS domain (IPR000014)			
AMIS_64560	497	Two-component system sensor histidine kinase (IPR050428)	AMIS_64570	233	Signal transduction response regulator, receiver domain (IPR001789); OmpR/PhoB-type DNA-binding domain (IPR001867)
AMIS_64930 (*hhkA*)	685	Histidine kinase domain (IPR005467); Signal transduction response regulator, receiver domain (IPR001789); GAF-like domain superfamily (IPR029016); PAS domain (IPR000014)			
AMIS_65210	402	Sensor histidine kinase two-component system (IPR050482)	AMIS_65220	219	Signal transduction response regulator, receiver domain (IPR001789); Transcription regulator LuxR, C-terminal (IPR000792)
AMIS_65550	380	Sensor histidine kinase two-component system (IPR050482)	AMIS_65540	209	Signal transduction response regulator, receiver domain (IPR001789); Transcription regulator LuxR, C-terminal (IPR000792)
AMIS_66020	538	Hisitidine kinase domain (IPR005467); Cyclic nucleotide-binding domain (IPR000595)	AMIS_66030	552	Signal transduction response regulator, receiver domain (IPR001789); FAD/NAD(P)-binding domain (IPR023753)
AMIS_66180	401	Sensor histidine kinase two-component system (IPR050482)	AMIS_66170	224	Signal transduction response regulator, receiver domain (IPR001789); Transcription regulator LuxR, C-terminal (IPR000792)
AMIS_66270	386	Sensor histidine kinase two-component system (IPR050482)	AMIS_66280	222	Signal transduction response regulator, receiver domain (IPR001789); Transcription regulator LuxR, C-terminal (IPR000792)
AMIS_67670	426	Sensor histidine kinase two-component system (IPR050482)	AMIS_67660	211	Signal transduction response regulator, receiver domain (IPR001789); Transcription regulator LuxR, C-terminal (IPR000792)
AMIS_68370	405	Two-component system sensor histidine kinase (IPR050428)	AMIS_68380	224	Signal transduction response regulator, receiver domain (IPR001789); OmpR/PhoB-type DNA-binding domain (IPR001867)
AMIS_69810	428	Sensor histidine kinase two-component system (IPR050482)	AMIS_69820	219	Signal transduction response regulator, receiver domain (IPR001789); Transcription regulator LuxR, C-terminal (IPR000792)
AMIS_69920	659	Histidine kinase domain (IPR005467); Signal transduction response regulator, receiver domain (IPR001789); PAS domain (IPR000014)			
AMIS_72730	527	Hisitidine kinase domain (IPR005467); HAMP domain (IPR003660)	AMIS_72740	237	Signal transduction response regulator, receiver domain (IPR001789); OmpR/PhoB-type DNA-binding domain (IPR001867)
AMIS_72970	515	Histidine kinase domain (IPR005467); PAS fold-4 (IPR013656); Histidine kinase PdtaS, GAF domain (IPR022066)			
AMIS_74770	1,189	Histidine kinase domain (IPR005467); HAMP domain (IPR003660); Nitrate/nitrite sensing protein (IPR013587)			
AMIS_75240	414	Histidine kinase domain (IPR005467); GAF domain (IPR003018)			
AMIS_75990 (*phoR*)	426	Histidine kinase domain (IPR005467)	AMIS_75980 (*phoP*)	227	Signal transduction response regulator, receiver domain (IPR001789); OmpR/PhoB-type DNA-binding domain (IPR001867)
AMIS_76100	491	Histidine kinase domain (IPR005467); PAS domain (IPR000014)			
AMIS_78700	1,102	Histidine kinase/HSP90-like ATPase domain (IPR003594); HAMP domain (IPR003660); Nitrate/nitrite sensing protein (IPR013587)			
AMIS_79010	439	Histidine kinase domain (IPR005467); HAMP domain (IPR003660)	AMIS_79020	228	Signal transduction response regulator, receiver domain (IPR001789); OmpR/PhoB-type DNA-binding domain (IPR001867)
AMIS_79460	356	Histidine kinase domain (IPR005467); HAMP domain (IPR003660)	AMIS_79450	219	Signal transduction response regulator, receiver domain (IPR001789); OmpR/PhoB-type DNA-binding domain (IPR001867)
AMIS_79540	408	Histidine kinase/HSP90-like ATPase domain (IPR003594); Signal transduction histidine kinase, subgroup 3, dimerization and phosphoacceptor domain (IPR011712)	AMIS_79550	215	Signal transduction response regulator, receiver domain (IPR001789); Transcription regulator LuxR, C-terminal (IPR000792)
AMIS_79610	404	Sensor histidine kinase two-component system (IPR050482)	AMIS_79620	220	Signal transduction response regulator, receiver domain (IPR001789); Transcription regulator LuxR, C-terminal (IPR000792)
AMIS_79710	377	Histidine kinase/HSP90-like ATPase domain (IPR003594); Signal transduction histidine kinase, subgroup 3, dimerization and phosphoacceptor domain (IPR011712)	AMIS_79700	219	Signal transduction response regulator, receiver domain (IPR001789); Transcription regulator LuxR, C-terminal (IPR000792)
AMIS_79760	439	Sensor histidine kinase two-component system (IPR050482)	AMIS_79770	222	Signal transduction response regulator, receiver domain (IPR001789); Transcription regulator LuxR, C-terminal (IPR000792)
AMIS_80230	401	Histidine kinase/HSP90-like ATPase domain (IPR003594); Signal transduction histidine kinase, internal region (IPR010559)	AMIS_80240	245	Signal transduction response regulator, receiver domain (IPR001789); LytTR DNA-binding domain (IPR007492)
AMIS_80260	345	Histidine kinase domain (IPR005467); HAMP domain (IPR003660)	AMIS_80250	230	Signal transduction response regulator, receiver domain (IPR001789); OmpR/PhoB-type DNA-binding domain (IPR001867)
AMIS_80400	387	Histidine kinase domain (IPR005467)			

[a] Orphan HKs with or without response regulator receiver domains are shown in blue or orange, respectively.

[b] Conserved domains in each gene product identified by *in silico* search using the InterPro ver. 101.0 (https://www.ebi.ac.uk/interpro/) are shown. The threshold of the *E* value is 0.01.
